# Signaling within Allosteric Machines: Signal Transmission Pathways Inside G Protein-Coupled Receptors

**DOI:** 10.3390/molecules22071188

**Published:** 2017-07-15

**Authors:** Damian Bartuzi, Agnieszka A. Kaczor, Dariusz Matosiuk

**Affiliations:** 1 Department of Synthesis and Chemical Technology of Pharmaceutical Substances with Computer Modelling Lab, Medical University of Lublin, 4A Chodźki Str., Lublin PL20093, Poland; agnieszka.kaczor@umlub.pl (A.A.K.); dariuszmatosiuk@umlub.pl (D.M.); 2 School of Pharmacy, University of Eastern Finland, Yliopistonranta 1, P.O. Box 1627, Kuopio FI-70211, Finland

**Keywords:** GPCRs, signaling, allostery, protein dynamics

## Abstract

In recent years, our understanding of function of G protein-coupled receptors (GPCRs) has changed from a picture of simple signal relays, transmitting only a particular signal to a particular G protein heterotrimer, to versatile machines, capable of various responses to different stimuli and being modulated by various factors. Some recent reports provide not only the data on ligands/modulators and resultant signals induced by them, but also deeper insights into exact pathways of signal migration and mechanisms of signal transmission through receptor structure. Combination of these computational and experimental data sheds more light on underlying mechanisms of signal transmission and signaling bias in GPCRs. In this review we focus on available clues on allosteric pathways responsible for complex signal processing within GPCRs structures, with particular emphasis on linking compatible in silico- and in vitro-derived data on the most probable allosteric connections.

## 1. Introduction

Allostery is one of the most essential properties of proteins, making them versatile machines capable of complex function. The term, originating in Greek words ἄλλος (allos—other) and στερεὀς (stereos—object) was originally used to describe events occurring in biological ternary complexes, where a ligand binding to ‘other site’ in a protein affects action of a ligand bound at ‘orthosteric’ site, i.e., the one responsible for main protein function. In the present literature however, definition of allostery is frequently broadened to encompass all spatially distant effects of protein excitation at a particular site, even if the investigated complex is composed of only two elements. Deeper insight into principles governing function of various proteins frequently reveals sophisticated allosteric mechanisms involved, and in many cases, proteins that were thought to perform their function in a simple and straightforward way show their true, more complicated face. G protein-coupled receptors (GPCRs) are a good example of such a situation. These receptors play an essential role in signaling in vertebrates, and can be also found in invertebrates, fungi, plants, or even protozoa. They are responsible for sensing a broad variety of impulses, including e.g., neurotransmitters, hormones, ions, proteases, olfactory and visual stimuli. There are ca. 800 genes encoding GPCRs in human genome, which makes them the largest family of human receptor proteins, and one of the largest protein families in general [1]. Unsurprisingly, this makes them one of the main drug targets—ca. 30% of currently available medicines are targeting GPCRs [2]. All these receptors share a common fold of seven transmembrane helices connected by three intracellular and three extracellular loops. Most of them share also a number of conserved sequence motifs, and it is suggested that their activation and modulation might be governed by similar mechanisms. GPCRs were once thought to be simple signal relays, receiving extracellular stimuli and transmitting them to a defined species of G proteins in the intracellular space. Notably, even according to this deprecated model, signal transmission within GPCRs could be regarded as an allosteric process, since ligand binding in the extracellular binding pocket was supposed to induce changes at the distant, intracellular site. Recent years, however, brought significant advance in understanding GPCRs function. Today it is known, that they can receive signals and process them, with the signaling outcome depending on the actual structure of a ligand, as well as on presence of additional allosteric factors, like allosteric small molecules, membrane components, ions, or even other GPCRs (in receptor dimers/oligomers). A single GPCR can recognize various ligands, and depending on their nature, it can activate various intracellular signaling cascades, involving different G protein heterotrimers, arrestins or scaffolding proteins. Moreover, it is known, that these pathways can be activated separately by appropriate ligands—this phenomenon is called ‘functional selectivity’. Therefore, it can be concluded that in order to activate different signaling cascades, functionally selective ligands have to activate different intra-protein signal transmission pathways, which in turn suggests that there are more than one way of signal processing in GPCRs. This means that they should rather be considered as complex allosteric machines capable of processing the signal within their structure and returning different responses to different stimuli. In this review, we focus on allosteric pathways responsible for such complex signal processing within GPCRs structures.

### 1.1. Allosteric Pockets in GPCRs

Knowledge of possible allosteric pockets in GPCRs is an important clue. Such pockets are most probably located at the end or along an allosteric pathway, and since many GPCRs share the seven transmembrane (7TM) scaffold as well as numerous conserved sequence motifs, such pathways might be present in many related receptors. In contrast to the orthosteric pockets in receptor structures, allosteric binding sites are often less defined and can be located in various areas. Since native stimuli usually come from the extracellular side, orthosteric pockets are also located at this side of the receptor, while location of the allosteric sites is not so obvious. Allosteric ligands can bind to every receptor surface, including extracellular, intracellular [3,4] and transmembrane regions [5,6,7]. Notably, not all allosteric sites can be identified at the first sight in the static X-ray structures or homology models. Such pockets are frequently located in less ordered regions than orthosteric ones, and slight conformational changes can easily affect their shape or charge distribution. Therefore, it is possible that a particular pocket is hidden in a static X-ray or homology model snapshot of a protein. Such allosteric sites are known as ‘hidden pockets’ [8,9,10,11,12]. As a consequence, cavity search performed on a static GPCR structure may miss a potent allosteric site. On the other hand, lack of a cavity in a putative allosteric site location, e.g., resulting from hot-spot calculation [13] doesn’t mean that such location should be rejected.

The number of methods helpful in allosteric site prediction were reviewed recently [14,15]. In particular, the AlloSteric Database serves as a source of knowledge on allosteric sites known so far [16]. Due to a considerable conservation of 7TM scaffold and possible common activation mechanisms, location of already identified allosteric sites present in related receptors may be helpful. Remarkably, some of the allosteric pockets were identified or suggested by X-ray crystallography. These include the allosteric pockets in the glutamate receptors [17,18,19], M_2_ muscarinic receptor [20], CCR5 chemokine receptor [21], a putative pocket in the A_2A_ adenosine receptor [22], and, perhaps the most intriguing, allosteric sites located at the intracellular side of CCR2 [3] and CCR9 [4] chemokine receptors. In general, the most frequent location of allosteric pockets depends on the GPCR family. In class C GPCRs, the allosteric pocket is usually located inside the transmembrane bundle, while orthosteric ligands bind to a developed extracellular regions. Meanwhile, in Class A receptors it’s the orthosteric pocket that is located relatively deep inside the transmembrane bundle, while modulators bind to the more exposed regions. One broadly known exception is the allosteric sodium ion, which was identified in number of GPCR X-ray structures [23,24,25,26], and was found to be located at the conserved Asp 2.50 deep in the 7TM bundle. The present state of knowledge on the role of sodium ion and details of its binding site were extensively described recently [27].

### 1.2. Molecular Switches and Large-Scale Motions

It is broadly known that all the GPCRs share the common structural scaffold of seven transmembrane helices. Moreover, while there are hundreds of GPCRs, their excitation results in activation of G proteins, which are significantly less diverse. Although 20 variants of Gα, 6 variants of Gβ and 12 variants of Gγ subunits could allow for large number of combinations, not all configurations are possible. Furthermore, it is the Gα subunit that bears most responsibility for coupling with receptors, and on the basis of the Gα subunit type all the heterotrimers can be classified into as few as four main families. Therefore, there is an apparent discrepancy between number of G protein heterotrimers and number of known GPCRs. It can be concluded that various receptors can couple with particular G proteins. Common structural scaffold and activation of very similar intracellular coupling partners clearly suggests that some common mechanisms can be in play. It is the most apparent in case of class A GPCRs, which share a number of very conserved sequence motifs, believed to play a role of molecular switches. Most of these switches and their action were extensively reviewed by Trzaskowski et al. [28], and some interesting information can be found in earlier works [29,30]. The most important putative molecular switches are the ionic lock involving the conserved DRY motif at third transmembrane helix (TM3) and 6.30 and/or 6.34 residue (Ballesteros-Weinstein notation [31]) from TM6, 3–7 lock switch constituted by 3.28 and 7.43 residues from TM3 and TM7, respectively, the transmission switch involving the Trp residue from the conserved CWxP motif, tyrosine toggle switch involving Tyr 7.53 from NPxxY motif [28], and the hydrophobic barrier, preventing water from establishing a continuous chain through an inactive receptor and broken upon activation [32]. Recently, a new possible switch was identified by Venkatakrishnan et al. [33]. Through analyzing contacts between side chains in all possible X-ray structures of class A GPCRs, they noticed that in all inactive structures the 3x46 residue (‘x’ instead of dot indicates the GPCRdb numbering scheme [34] used by the authors in the original report, largely analogical to the Ballesteros-Weinstein scheme) interacts with the 6x37 side chain, while in all active structures it prefers contact with the 7x53 residue from the NPxxY conserved motif. Another detailed study on molecular switches, performed by Lee et al., on the A_2A_ adenosine receptor, resulted in identification of 10 binary switches that characterize distinct activation states [35,36]. Among these binary switches, role of the rotameric state of the Trp 6.48 from the conserved CWxP motif turned out to be the most pronounced.

Change in conformation of one or more molecular switches is believed to trigger large-scale conformational rearrangements in the entire receptor protein. The most recognized of them is the outward movement of the intracellular part of the TM6 [37,38,39,40,41]. Other changes believed to be involved in activation are translocation of TM5 [42] and/or TM3, as well as rearrangements in TM7 [41,43]. However, exact causal relationship between triggering the switches and conformational rearrangement is difficult, and despite the intensive efforts, manifested in increasing number of reports on allosteric intra-protein signaling pathways in GPCRs, exact mechanisms remain largely unknown.

## 2. Allosteric Pathways within Single Receptor Proteins

Identification of binding pockets or molecular switches provides an important insight into GPCR function and is very helpful in design of precise pharmacological tools or potential novel drugs. However, understanding of exact relationships between binding sites and effector sites would greatly facilitate the design of functionally selective, efficient substances. Notably, distances between identified binding pockets, molecular switches and effector sites are significant. This clearly suggests that the signal induced in the binding site has to be propagated through the protein structure in an allosteric way. Investigation of the allosteric signaling pathways is even more problematic than finding the binding site or a switch, since signal propagation is likely to involve a series of subtle, hardly intangible events that eventually result in a large-scale rearrangement. Although these eventual conformational changes can be investigated experimentally, they are a result of the allosteric signal transmission, the mechanisms of which remain elusive. A number of attempts of the pathways elucidation was undertaken in recent years. In many cases, combinations of computational and experimental approaches turned out to be beneficial.

### 2.1. Allosteric Pipelines in 6th and 7th Transmembrane Helices of Class A GPCRs

Notably, some GPCR regions keep being suggested as important or crucial for proper receptor function in numerous reports in the field. In particular, transmembrane helices 6th and 7th (TM6 and TM7, respectively) are among the most frequently mentioned GPCR fragments. TM6 was suggested to play an important role in GPCR activation relatively long time ago. X-ray structures of active-state receptors coupled with G proteins or nanobodies have proven that TM6 undergoes large-scale rearrangement, involving rigid-body movement of the helix, which eventually increases the distance between intracellular parts of TM6 and TM3, preparing room for G protein binding. TM7 in turn is known for its conserved NPxxY motif, suspected to play role in rearrangement of hydrogen bonding network upon activation.

Recently, some computational and experimental efforts provided deeper insight into exact mechanisms responsible for TM6 rearrangement, and into a role of the NPxxY motif in signal processing. These contributions provide more and more pieces of the puzzle, and collecting them together allows one to see the greater picture emerging. In a very inspiring computational work of Bhattacharya and Vaidehi [44], authors calculated correlations of torsion angles of amino acid side chains in microsecond-scale simulations of β_2_ adrenergic receptor in its active, inactive, and intermediate (agonist-bound but deprived of G protein) states. They used the mutual information (MI) method, which is one of known methods of finding intra-protein relationships [14,45]. First important conclusion drawn from their calculations was that indeed, there is an allosteric coupling of the distant residues in GPCRs. MI calculated for neighboring side chains presented obvious correlations, which quickly decreased with increasing distance. However, correlations started to rise above a particular border distance to approx. 60 Å. This clearly indicated that there are conformational changes that are propagated through the protein and can be calculated with mutual information approach. More detailed calculations allowed for finding the regions with largest mutual information, as well as for identification of pathways connecting these regions. These pathways were found via maximizing the mutual information of neighboring components of the pathway while minimizing its length. A number of individual pathways presented significant overlap and could be grouped into clusters. The clustering resulted in identification of allosteric pipelines, i.e., routes within the receptor concentrating essential part of internal allosteric signaling. Interestingly, a number of most important allosteric pipelines were concentrated at TM6 and TM7. Intensity of signaling through these pipelines varied between activation states, which could be reasonably attributed to activation events. The most evident pathway of allosteric communication in the inactive receptor was noticed at the TM6. It connects extracellular and intracellular parts of the receptor with significant mutual information values across the entire helix. However, such pipeline was not detected in partially active or active receptor conformations. According to these results, agonist binding seems to unharness the correlation of movements in the extracellular and intracellular parts of the receptor, which removes restriction of TM6 movement. This would be consistent with computational study of Miao et al., who concluded from their accelerated MD simulations that communication in the intracellular part of M2 muscarinic receptor is much weaker during activation [46]. The binding pocket seems to gain the role of a command center, with number of new pipelines starting at the site. Moreover, new significant pipelines located at TM7 are observed in the active and partially active conformations. This notice is especially important considering the role of TM7 in the arrestin recruitment. It is known that the GPCR-arrestin coupling involves C-terminal part of the receptor, as well as third intra-cellular loop (ICL3). Recently it was proven that interaction with intracellular loops is not inevitable for arrestin recruitment, and it is the C-terminus that is essential for such interactions [47]. Residues located at TM7 were also suggested to play an important role in functional selectivity, e.g., in a preference of the β_2_ adrenergic receptor to couple with β-arrestin, Gi or Gs proteins [48], as well as in mechanisms of functional selectivity and allosteric modulation of opioid receptors [49,50].

Summarizing, the identification of allosteric pipelines at β_2_ adrenergic receptor through MI calculation and subsequent pathway clustering suggests that agonist binding results in disruption of the previous allosteric signaling through TM6 and establishing the new signaling network, involving the orthosteric site as the main command center, with TM7 as its main component. The first event releases TM6 from restraints and allows for its outward movement, the second establishes ligand-dependent control of the orthosteric site over the intracellular part of TM7, which throws some light on the mechanisms of functional selectivity.

#### 2.1.1. The Role of 7.35 and 7.53 Residues

**Figure 1:**
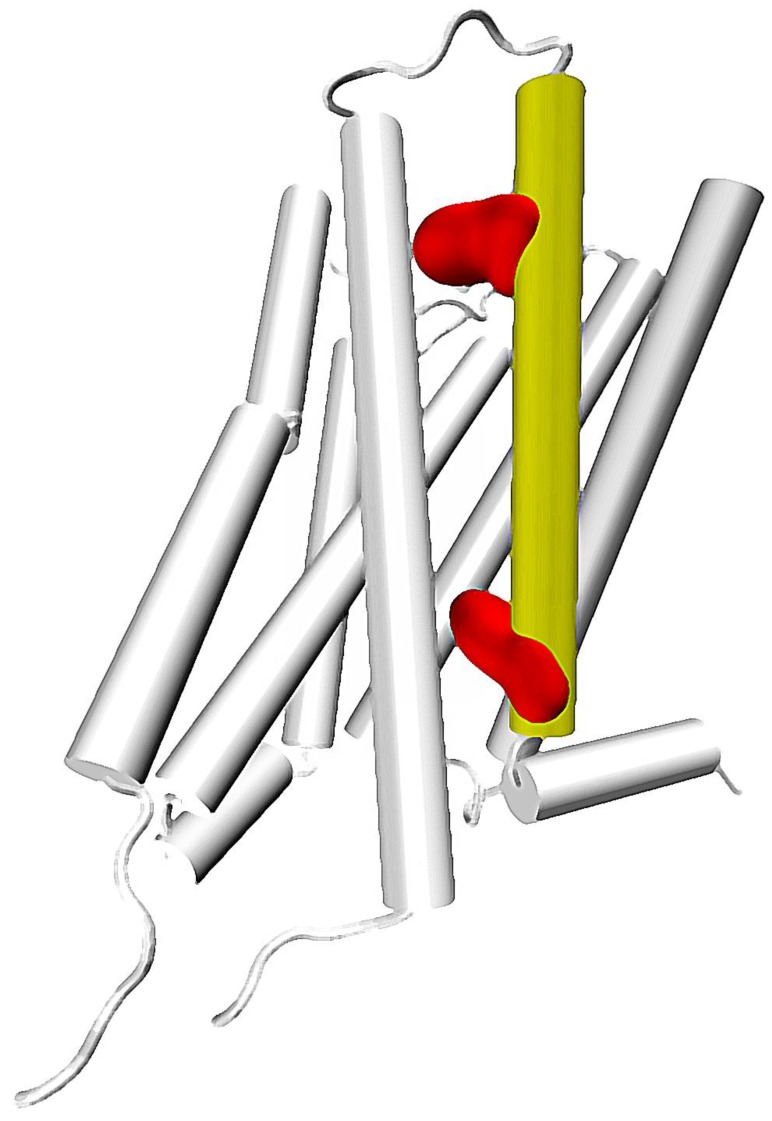
A G protein-coupled receptor with 7th transmembrane helix colored in yellow. 7.35 and 7.53 residues marked with red (upper and lower, respectively).

The calculated correlations in motions and putative role in functional selectivity described above are well reflected in experiments. In particular, identification of a particular residue located at the height of the orthosteric binding pocket at TM7—7.35 residue (numbering according to the Ballesteros-Weinstein residue notation [31])—as one of main sites responsible for inducing allosteric communication can be connected with number of other important reports. Recently, Woo et al., have proven that mutation of Tyr 7.35 into Phe in β_2_ adrenergic receptor can greatly affect the selective activation of Gi and Gs proteins. This results in a conclusion, that selectivity of β_2_ receptor toward one of these G proteins is regulated not only by phosphorylation patterns, but also directly by the allosteric signal induced in the binding pocket. The study of Bock et al., suggested importance of Trp 7.35 in Gi/Gs selectivity of the muscarinic M_2_ receptor [51]. The same residue was found to be essential in the M_2_ receptor interactions with its allosteric modulator LY2119620, which is well visible in X ray structures of the complex [20]. In turn, Hothersall et al., examined the role of Trp 7.35 together with Tyr 7.43 in μ opioid receptor (MOR) by mutating these residues to Ala and Phe, respectively. Their results confirmed the role of both residues in signaling bias of various ligands [50]. This is in line with computational results obtained by Schneider et al., who identify the Trp 7.35 residue as one of the main sites responsible for transmission of the signal induced by a G protein-biased agonist TRV130, but not in signaling resulting from binding of morphine, which presents lesser bias toward G proteins [52]. Notably, in a computational study of Bartuzi et al., performed on MOR, the rotameric transition of Trp 7.35 is suggested to affect conformation of a distant Tyr 7.53 residue through altered bending and rotation of the TM7. This would be in line with the mentioned calculations of Bhattacharya and Vaidehi, since both Trp 7.35 and Tyr 7.53 are located along one of the main allosteric pipelines suggested by them (Figure 1). Moreover, the latter residue was found to participate in one of the three most widespread contacts characteristic for GPCR activation, together with amino acids at the 3 × 46 and 6 × 37 locations [33]. The already mentioned recent study of Venkatakrishnan et al., presented, that the 3 × 46 residue tends to directly interact with the one at the 6 × 37 position, but loses this contact upon activation and establishes new interaction with 7 × 53 residue [33].

#### 2.1.2. TM2, TM7 and the Allosteric Sodium Ion

**Figure 2:**
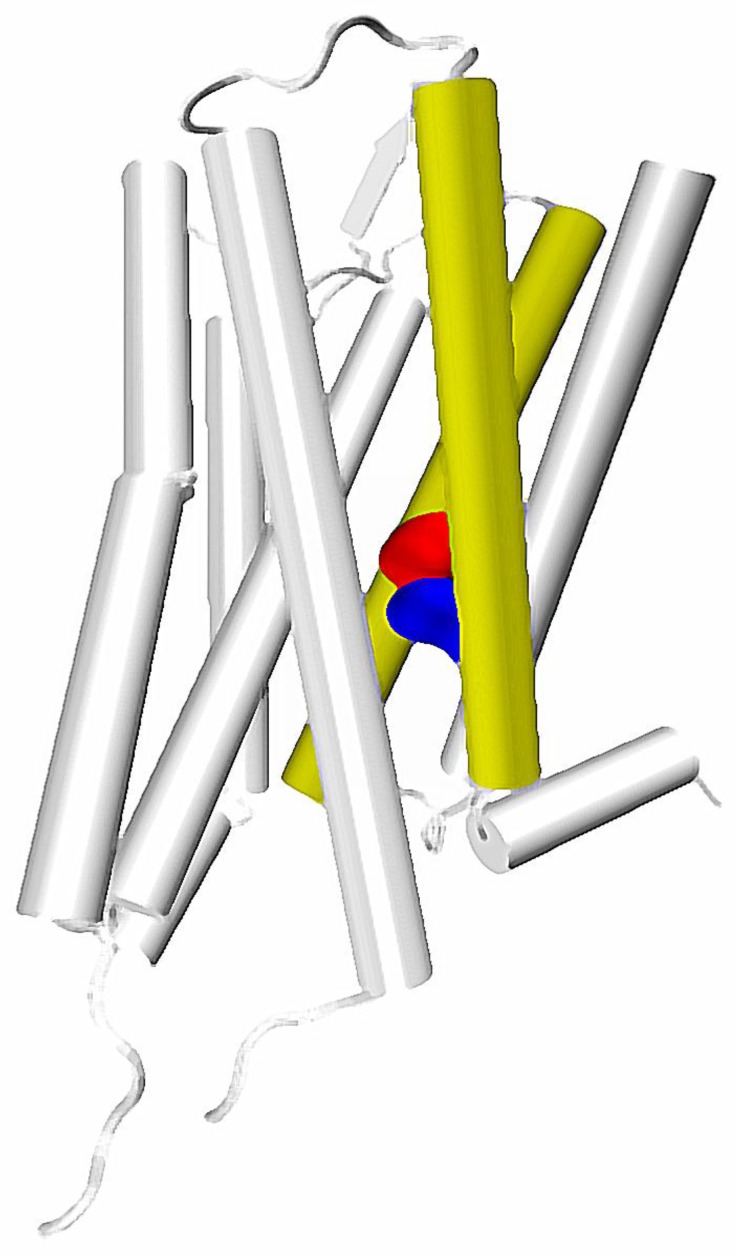
Location of TM2 and TM7 in a GPCR structure. Key residues: 2.50 and 7.49 marked with red and blue, respectively.

Interestingly, in the fully-activated structure, according to the mentioned study of Bhattacharya and Vaidehi, the pathway involving the TM7 starts in the extracellular part of TM2, goes down along this helix and jumps to the TM7 at the height of D2.50/N7.49, which are suggested to constitute the allosteric binding site for sodium ions (Figure 2). In turn, it was suggested by experimental work that sensitivity of a GPCR to the presence of sodium ions is connected with G protein/arrestin signaling bias [27], which further supports the hypothesis of a special role of the region in allosteric signaling within GPCRs.

The top of TM2/TM7 was recently suggested by two independent studies to be a part of putative allosteric pocket in opioid receptors [49,53]. One of these studies focuses on an allosteric modulator BMS986122, which was found to affect ligands ‘sensitive’ to presence of the allosteric sodium ion [54]. Some other computational works suggest that Asp 2.50, which is responsible for binding of the allosteric sodium, can be considered together with neighboring residues from TM1 and TM7 as an important ‘microswitch’ involved in activation of A_2A_ receptor [35]. In turn, the already mentioned study of Hothersall et al., proves that mutation of some residues in the δ opioid receptor, located at the TM7, i.e., Trp 7.35 and Tyr 7.43 results in a change in the ligand signaling bias [50]. Notably, mutation of an Asn 7.49 into Ala residue in the δ opioid receptor turns known opioid antagonists into β-arrestin biased agonists, and it is known from the high-resolution X-ray structure of this receptor, that the 7.49 residue directly interacts with the allosteric sodium [26]. Considering all these premises together leads to a suspicion, that presence of the allosteric sodium greatly affects the conformation of TM7, which in turn affects the signaling bias. Furthermore, since sodium-interacting residues are present in both TM2 and TM7, allosteric signals affecting the ligand bias can originate from the top of both TM2 and/or TM7, which is still consistent with tracks of allosteric pipelines identified by Bhattacharya and Vaidehi [44].

#### 2.1.3. Role of TM5/TM6 Interactions in Signal Transmission

**Figure 3:**
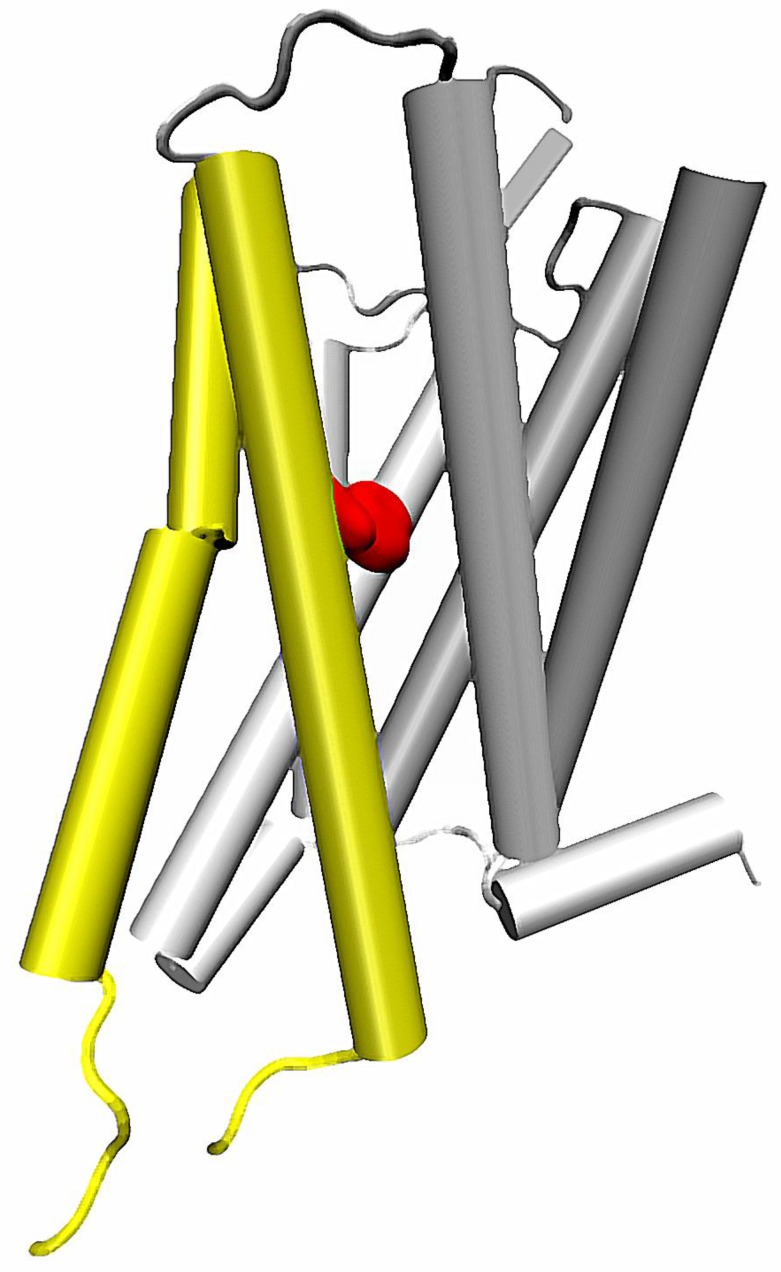
Location of 5th and 6th helices in a GPCR structure. Conserved Trp 6.48 residue marked with red.

Importantly, the sodium-related β-arrestin/G protein signaling bias can be also affected by mutations in other helices. One of the most recent reports on such possibility concerns mutation of the conserved Trp 6.48 residue from the CWxP motif on the TM6 [55] (Figure 3). First, the molecular dynamics of δ opioid receptor in complex with agonist, antagonist and sodium ion in various configurations indicated that presence of the allosteric sodium affects the χ_1_ dihedral of Trp 6.48. Two most frequent rotameric states of the side chain were identified, and in particular they were attributed to receptor conformations in presence or absence of the Na^+^ at the conserved Asp 2.50. Moreover, the initial conclusion on relationship between sodium and Trp 6.48 was confirmed by in vitro examination. It turned out, that mutation of the tryptophan into any of the tested amino acids, i.e., Ala, Phe, Asp and Leu resulted in abolishment of β-arrestin recruitment upon stimulation by DADLE and significant reduction of the recruitment by another tested agonist, BW373U86, with only minimal effect on G protein mediated signaling. These data further support the hypothesis on involvement of Na^+^ in the regulation of signaling bias in GPCRs, and suggest another pathway of the signal propagation from the allosteric sodium binding site. Detailed analysis of MD simulations suggest, that conformation of Trp 6.48 is correlated with orientation of TM5 relative to other helices [55]. In turn, the altered conformation of TM5 induced by a rotameric transition of Trp 6.48 seems to be propagated to other regions of the TM6.

Another involvement of TM5 and TM6 was also recently investigated by Ozgur et al. [56]. They computationally studied the β_2_ adrenergic receptor together with the intracellular loop 3 (ICL3), which is known to be problematic and frequently replaced by more stable protein fragments in X-ray crystallography studies in order to facilitate crystallization. It is also usually omitted in a molecular dynamics studies, due to its less ordered character. ICL3 links TM5 and TM6 on the intracellular side of GPCRs, and the study of Osgur et al., suggests that its conformation is in reciprocal relationship with arrangement of these helices. In particular, the inward tilt of TM5 and TM6 resulting from the outward tilt of their extracellular parts was found to promote the packing of ICL3 into a stable conformation, closing the entrance for G proteins and therefore named the ‘very inactive state’ conformation.

### 2.2. Role of TM3 in Allosteric Signal Transmission

Generality of mentioned calculations performed by Bhattacharya and Vaidehi on β_2_ adrenergic receptor was recently validated by Bhattacharya et al., with a larger set of calculations performed on inactive-state X-ray structures of eight class A GPCRs [57]. Using the same method, which has gained a name ‘Allosteer’, they analyzed dynamics of protease activated receptor-1, A_2A_ adenosine receptor, β_1_ and β_2_ adrenergic receptors, M_2_ and M_3_ muscarinic receptors, D_3_ dopamine receptor and H_1_ histamine receptor. In this recent work, they found that the allosteric pipeline leading through TM7 is conserved in all investigated receptors. However, they also found that a number of allosteric hubs conserved across these receptors involve regions of TM3. Moreover, another conserved allosteric pipeline was identified. The pipeline starts at the EC part of TM2, ends at IC part of TM5 and TM6, and its large part involve TM3.

In contrast to TM6, TM3 is known to be relatively stable and not to undergo large-scale rearrangements during activation. However, the data presented by Bhattacharya et al., suggest that there are correlated motions of residues constituting TM3, and this feature is conserved among class A GPCRs. Considering this finding together with present knowledge on 3–7 and ionic lock switches [28] as well as with results of other reports, e.g., the already mentioned paper of Venkatakrishnan et al. [33] or a work of Gregory et al. [58] which suggests a number of molecular switches involving residues located at TM3, one can come to the conclusion that TM3 plays an essential role in propagation of allosteric signals through GPCRs. In particular, the work of Venkatakrishnan et al., sheds some light on how the signal can be transmitted, spotting the 3 × 46 residue as one of putative switches participating in signal propagation and in altering network of interactions between TM3 and TM6/TM7. On the other hand, Gregory et al., found that the 3.28 residue regulates signaling bias in M2 muscarinic receptor. Notably, the latter residue was also identified by Bhattacharya et al., as one of the allosteric hubs [57].

### 2.3. Water-Mediated Pathways

The studies described above usually focus on the behavior of protein itself, i.e., amino acid conformations, correlations in side chains’ movements and large-scale rearrangements induced by these factors. Notably, some reports indicate that mechanisms of such events should not be considered without taking solvent molecules into account. For instance, participation of water molecules in the activation of rhodopsin was suggested by Sun et al. [59]. Their computational study involved molecular dynamics simulations of rhodopsin in inactive, constitutively active and Meta II states, followed by inhomogenous fluid theory calculations. This approach yielded very interesting results, compatible with most of already mentioned reports, and providing some new insights into mechanisms of some molecular switches. In particular, favorable hydration sites were found at the extracellular loops together with terminus, at the ligand binding pocket as well as at the NPxxY motif from the TM7. While the first two regions have some unusual properties in rhodopsin, e.g., a covalently-bound ligand and the binding pocket tightly covered with β-sheet, which makes these data less transferable to other family members, NPxxY motif is very conserved in GPCRs and frequently mentioned in previous sections as a crucial element of molecular switches and signaling pathways, so the observed behavior of functional water molecules in its neighborhood may be considered as a more general pattern. These water molecules were found to participate in hydrogen bonding between TM2, TM6 and TM7. Moreover, rearrangements in hydrogen bonding upon activation were observed.

Another study focused on rhodopsin, reported by Leioatts et al., utilized molecular dynamics simulations together with solid-state NMR and focused on the behavior of retinal in its binding pocket [60]. Although, as mentioned, the pocket is quite specific and unusual for GPCRs, they managed to observe some events of more general importance. At initial stages of activation, the conformation adopted by the ligand allowed for significant influx of water into the hydrophobic core of the receptor. Additionally, a conformational change at the conserved Trp 6.48 was observed. The alteration of water flux at the transmembrane bundle seems to play important role in activation of various GPCRs, including A2A adenosine receptor [61] and μ opioid receptor [62,63]. In the former, Lee et al., found that the water molecules inside the receptor bundle flow three times more slowly in the active state than in inactive state, and that some ultraslow water molecules are found in the active state in the nearness of microswitch residues at TM3, TM6 and TM7. In the latter receptor, Yuan et al., investigated the role of sodium ion in receptor activation [62]. They noticed that in presence of sodium ion at its allosteric site the number of water molecules inside the transmembrane bundle increased. Moreover, exchange of these molecules with extracellular water was faster in presence of agonists than in complexes with antagonists. These phenomena were later utilized by Bartuzi et al., in analysis of MD simulations of allosteric modulation of MOR [63], with results being consistent with the report of Yuan et al.

**Figure 4:**
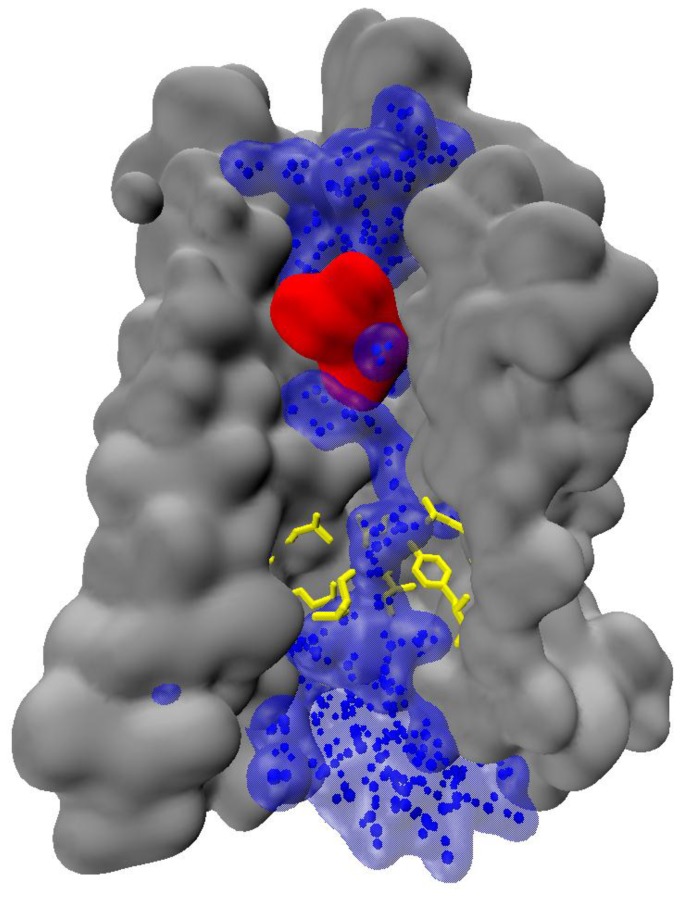
A continuous chain of water molecules spanning a GPCR (an exemplary snapshot from in-house trajectories of authors).

The above-mentioned observations indicate, that water can play significant role in signal transmission. Notably, water chain can span the entire activated receptor [32,63], reaching deeply buried receptor residues (Figure 4). Formation of the continuous water chain upon activation may significantly affect the internal hydrogen-bonding networks, and therefore, water can play significant role in signal propagation.

## 3. Signal Transmission between Dimer Subunits

### 3.1. Dimerization of GPCRs and Its Consequences for Drug Design

The classical ternary complex model, which assumes the interplay of three basic components: a receptor, an agonist and a G protein, served for a long time as description of GPCRs function [14]. In the light of this model, activation of a receptor is a result of interaction with an agonist, which leads to the activation of a specific G protein in the intracellular region that, in turn, initiates particular signaling cascades. However, experimental and computational studies have demonstrated that GPCR functioning and signaling can be much more complicated than this classical model predicts.

In particular, it was first thought that GPCRs function as monomeric entities. However, biophysical and biochemical methods (cross-linking experiments, BRET and FRET studies [64]) and molecular modeling approaches [65] (coarse-grained molecular dynamics simulations [66]) reporting negative and positive cooperativity provide more and more evidence that these receptors form functional homomers and heteromers [67,68,69]. The hypothesis about dimerization/oligomerization of GPCRs was proposed by Fuxe at al. in the 1980s [70,71]. The studies on dimerization of GABA_B_ receptors supplied the first proofs for this hypothesis. Nowadays, it is widely accepted that family C GPCRs (e.g., GABA_B_ receptors, metabotropic glutamate receptors) form constitutive dimers. In the year 2000 the data about dimerization of µ and δ opioid receptors [72] and dopamine D_1_ and adenosine A_1_ receptors [73] were published. These observations resulted in a long discussion whether rhodopsin-like GPCR dimers are also constitutive and required for G protein activation [69]. The studies in which monomeric entities were trapped into nanodiscs clearly demonstrated that this is not the case [74,75,76]. Moreover, monomeric rhodopsin in solution activated its G protein—transducing—at the diffusion limit [77]. The functionality of GPCR monomers has been also demonstrated when isolated monomeric transmembrane domains of mGluR2 (family C GPCR) were able to fully activate G protein when directly activated by small synthetic ligands [78]. Moreover, a 1:1 stoichiometry has been shown as sufficient for rhodopsin–β-arrestin interactions [79,80]. Importantly, these experiments did not exclude that class A GPCR oligomers can be spontaneously formed in living cells, and raised the question of their functional significance [69].

Unique properties of GPCR oligomers result from allosteric mechanisms. Under cooperative dimer model [69] the two-state model supplies practical tools for analysis of ligands with dimers. In particular, it is important to note that binding of an agonist with one monomer modulates agonist affinity to the other unit of this dimer [81] which may have physiological consequences—for instance, a heterodimer of serotonin 5-HT_2A_ receptor and mGluR2 receptor is important in psychosis [82]. Similarly, the cases of cross-antagonism have been described: selective antagonist of one monomer may block agonist-induced signal transduction through the other monomer in a dimer [81]. Considering receptor heteromers as an example of allosteric modulation of GPCRs, there are cases of allosteric modulation by specific ligands of some receptors of not only affinity but also intrinsic efficacy of ligands for other receptors [69]. Moreover, there are reports concerning functional selectivity, where one of the protomers of the heteromer acts as an allosteric modulator that “forces” the other receptor protomer to signal predominantly through a distinct signaling pathway [69].

The GPCR heteromers with their allosteric properties constitute a new signaling and pharmacological entities which are novel and promising drug targets. In particular, the capability of one receptor to behave as an allosteric modulator of its dimer partner opens possibility of targeting the receptor acting as conduit of the allosteric modulation with selective ligands [69]. It can be exemplified by the fact that adenosine A_2A_ receptor antagonists enhance the affinity of the dopamine D_2_ receptor for dopamine, which can be a strategy for the treatment of Parkinson’s disease. Indeed, it has been demonstrated that the therapeutic index of L-dopa is increased with adenosine A_2A_ receptor antagonists [83]. Thus, targeting GPCR heteromers can help to overcome selectivity problem which is a major issue with drugs targeting many receptors. GPCR oligomers, due to their restricted tissue distribution, could also provide a new source of drug specificity.

### 3.2. Mechanisms of Signal Transduction through Dimers

The biochemical fingerprint of a heteromer includes pharmacological, signaling, and trafficking properties [84]. The majority of research aimed to investigate the biochemical fingerprint of a heteromer applied heterologous cells engineered to express recombinant receptors [79]. The benefits of this approach are: (i) it enables control of the expression level of GPCR protomers; (ii) cells that express individual protomers can be used as controls; and (iii) it addresses the question of whether the expression of the two receptors is sufficient to reveal the biochemical fingerprint of the heteromer in a native tissue [79].

#### 3.2.1. Modulation of Ligand-Binding Properties

As it has been already mentioned, studies using radioligand-binding assays have demonstrated that GPCR heteromerization can change the binding properties of protomer-selective ligands [84]. These changes may include an increase or decrease in binding affinity of such compounds. Accordingly, George et al. [85] showed that when µ and δ opioid receptors were co-expressed, the highly selective synthetic agonists for each had reduced potency and altered rank order, whereas endomorphin-1 and Leu-enkephalin had enhanced affinity, suggesting the formation of a novel binding pocket. Furthermore, Kabli et al. [86] demonstrated that δ-opioid agonists displaced µ-agonist binding with high affinity from µ-δ heteromers, but not µ receptor homomers, suggesting that δ-agonists occupy a novel µ-receptor ligand binding pocket within the heteromers. In this context, Baragli et al. [87] studied dopamine D_2_ receptor-somatostatin receptor (SSTR2) heterodimer and showed that agonist-induced heterodimerization was accompanied by increased affinity for dopamine. There are also reports about decrease or increase in binding affinity of ligands selective for one protomer without a change in affinity of ligands to the partner protomer [88,89]. Next, decrease or increase in the binding affinity of ligands that are selective for one protomer in the presence of a cognate ligand of the partner protomer have been also published. Dasgupta et al. [90] showed that in A_2A_-D_2_ cells the selective adenosine A_2A_ agonist 2-[p-(2-carboxyethyl)-phenethylamino]-5′-*N*-ethyl-carboxamido adenosine (CGS 21680) induced a 2–3-fold decrease in the affinity of dopamine D_2_ receptors for dopamine, as shown in competition experiments with dopamine versus the selective dopamine D_2_ antagonist [^3^H] raclopride. Albizu et al. [91] used radioligand binding and second messenger production assays to provide evidence for a functional crosstalk between 5-HT_2A_ receptors and D_2_ receptors in brain and in HEK293 cells. They found that D_2_ receptor activation increases the hallucinogenic agonist affinity for 5-HT_2A_ receptor and decreases the 5-HT_2A_ receptor-induced inositol phosphate production. Finally, there are also reports about positive or negative binding cooperativity using a combination of ligands that target each protomer [92,93,94]. As an example Sohy et al. [94] used a combination of luminescence complementation and bioluminescence resonance energy transfer assays to demonstrate the existence of hetero-oligomeric complexes composed of at least three chemokine receptors (CCR2, CCR5, and CXCR4). Moreover, they showed in T cells and monocytes that negative binding cooperativity takes place between the binding pockets of these receptors, demonstrating their functional interaction in leukocytes. They also proved that specific antagonists of one receptor (TAK-779 or AMD3100) lead to functional cross-inhibition of the others. Next, González et al. [93] showed that the production of both melatonin and serotonin by the pineal gland is regulated by a circadian-related heteromerization of adrenergic and dopamine D_4_ receptors. They suggested that through α(_1_B)-D_4_ and β_1_-D_4_ receptor heteromers dopamine inhibits adrenergic receptor signaling and blocks the synthesis of melatonin induced by adrenergic receptor ligands. In conclusion, all these results indicate that heteromerization may allosterically modulate receptor function.

#### 3.2.2. Modulation of Signaling Properties

Experiments applying signaling assays such as receptor mediated G protein activity, adenylyl cyclase activity, ERK1/2 phosphorylation, and β-arrestin-mediated signaling have demonstrated that heteromerization can change signal transduction [84]. The presence of one protomer may increase or decrease signaling by the partner protomer. As an example, Rosenfeld et al. [95] showed that heteromerization affects receptor signaling as the potency of the CB_1_ receptor ligand to stimulate G-protein activity is increased in the absence of δ opioid receptor, which leads to the conclusion that the decrease in CB_1_ receptor activity in the presence of δ opioid receptor can be explained, at least partially by heteromerization. Zhu et al. [96] proved that heterodimerization of β_1_ and β_2_ adrenergic receptors in intact cardiac myocytes creates a novel population of β adrenergic receptors with distinct functional and pharmacological properties, leading to increased signaling efficiency in response to agonist stimulation while silencing ligand-independent receptor activation, thereby optimizing beta-adrenergic modulation of cardiac contractility.

There are also reports that an agonist of one protomer signals through a different G protein subunit in cells expressing the heteromer compared with cells that express the homomer [84]. In this context Fan et al. [97] demonstrated that activation of the µ-δ opioid receptor heteromer resulted in preferential activation of Gα(z), whereas activation of the individually expressed µ and δ receptors preferentially activated Gα(i). In accordance with this results, Kabli et al. [98] showed that μ-δ opioid receptor heteromer activates the pertussis toxin-resistant Gα(z) protein following stimulation by the δ-agonist deltorphin-II whereas μ- and δ-receptors activate the pertussis toxin-sensitive Gα(i3) protein following stimulation by μ- and δ-agonists, respectively. Furthermore, Kern et al. [99] proved that formation of ghrelin receptor GHSR1a and D_2_ receptor heteromers allosterically modifies canonical D_2_ receptor signaling resulting in Gβγ subunit-dependent mobilization of [Ca²⁺] (i) independent of GHSR1a basal activity.

The signaling pathway activated by the heteromer and/or its localization can be different from that activated by each protomer [84]. It was studied by Rosenfeld and Devi [100] who proved that heterodimerization of µ with δ opioid receptors leads to a constitutive recruitment of β-arrestin2 to the receptor complex resulting in changes in the spatio-temporal regulation of ERK1/2 signalling. In another study, Lin and Trejo [101] demonstrated that PAR1-PAR2 dimers co-internalize and recruit β-arrestins to endosomes. Intriguingly, PAR1-PAR2 heterodimers seem to use a different interface for β-arrestin binding in comparison with receptor protomers. Furthermore, thrombin-activated PAR1-PAR2 heterodimers increase β-arrestin-mediated ERK1/2 activation in the cytoplasm, whereas activated ERK1/2 induced by the thrombin-activated PAR1 protomer redistributes to the nucleus. In recent study Bellot et al. [102] investigated if heterodimerization of presynaptic angiotensin II AT1 receptor and α_2C_-adrenergic receptor could underlie their functional cross-talk to control norepinephrine secretion. They demonstrated that dual agonist occupancy led to a conformation of the heterodimer different from that induced by active individual protomers and triggered atypical Gs-cAMP-PKA signalling.

There are also reports about a decrease in the potency of the agonist for one protomer. In this context Wang et al. [103] showed that heterodimerization of opioid receptor-like 1 and μ opioid receptors impairs the potency of μ receptor agonist. Furthermore, it has been concluded that there may be an increase or decrease in signalling with a combination of agonists to both protomers. As an example, Rios et al. [104] demonstrated that the simultaneous activation of µ opioid and CB_1_ cannabinoid receptors leads to a significant attenuation of the response seen upon activation of individual receptors, implicating a role for receptor-receptor interactions in modulating neuritogenesis.

As another example of modulation of signalling properties within GPCR heteromers, a combination of agonists for both protomers either fails to elicit signalling [105] or leads to novel signalling different from that of the individual protomers [102]. Pello et al. [105] showed that in immune cells expressing CXCR4 and δ opioid receprors, simultaneous addition of their ligands CXCL12 and [D-Pen2, D-Pen5] enkephalin does not trigger receptor function.

It was also demonstrated that an antagonist to one protomer blocks signalling by the other protomer. As an example, Carriba et al. [106] showed that, blockade of A_2A_ receptors counteracted the motor depressant effects produced by the intrastriatal administration of a cannabinoid CB_1_ receptor agonist, indicating that motor effects of cannabinoids depend on physical and functional interactions between striatal A_2A_ and CB_1_ receptors. Next, Sohy et al., investigated heterodimers formed by CCR2 and CXCR4 receptors and showed that specific antagonists of one receptor inhibit the binding of chemokines to the other receptor as a consequence of their heterodimerization, both in recombinant cell lines and primary leukocytes. It has been also proposed that the combined use of antagonists of both protomers blocks signalling from the heteromer. Leger et al. [107] used a combination of bivalirudin (hirulog) plus a novel PAR4 pepducin antagonist, P4pal-i1 in order to effectively inhibit aggregation of human platelets to even high concentrations of thrombin and prevented occlusion of carotid arteries in guinea pigs. Finally, there are findings that potentiation of signalling by one protomer occurs in the presence of non-signalling concentrations of ligands for the partner protomer [72,108].

In conclusion, it should be emphasized that, in most of the cases described above, these changes could arise from signalling crosstalk and not directly from allosteric receptor modulation [84]. Multiple studies have evaluated trafficking properties of protomers in the GPCR heteromers. These include reports about maturation of heteromers and their agonist-mediated internalization from the cell surface [84]. However, description of this phenomena is outside the scope of this review.

### 3.3. Molecular Aspects of Signal Transduction through GPCR Dimers

#### 3.3.1. Family C

Family C of GPCRs is unique within the entire superfamily and possesses distinct structural characteristics [109]. Receptors which belong to this family form constitutive dimers. In order to understand the conformational changes within such a dimeric receptor which are connected with agonist activation, Hlavackova et al. [110] studied the role of dimer formation in mGluR1 activation. They used FRET to evaluate inter- and intrasubunit conformational changes. Intrasubunit changes resulted in decrease in FRET, whereas intersubunit rearrangements resulted in increase in FRET, which supplied different signals with which to distinguish between these two processes [109,110]. Moreover, they used cotransfection of chimeric receptor subunits that were capable or incapable of G protein coupling to determine that only a single subunit assumes an active state in an mGluR1 receptor dimer [110].

Xue et al. [111] studied the metabotropic glutamate receptors and showed that structural changes at the dimer interface are linked to receptor activation. They demonstrated that the main dimer interface is built by a transmembrane helix 4 (TM4) and TM5 in the inactive state and by TM6 in the active state [111]. This key change in the dimer interface is essential for receptor activity as locking the TM4–TM5 interface prevents activation by agonist, while locking the TM6 interface leads to a constitutively active receptor [111]. Furthermore, Levitz et al. [112] showed that inter-subunit interactions between ligand-binding domains determine mGluR conformational gating dynamics and mediate receptor cooperativity and glutamate sensitivity.

A recent study by Kim et al. [113] concerns family C taste receptors TAS1R2/TAS1R3. They found that binding of agonists to Venus Flytrap Domains VFD2 of TAS1R2 leads to major conformational changes to form a TM6/TM6 interface between transmembrane domains (TMD) of TAS1R2 and TAS1R3, which is in accordance with the activation process known for the metabotropic glutamate receptor 2 homodimer [113]. They also demonstrated that the initial effect of the agonist is to pull the bottom part of VFD3/TAS1R3 toward the bottom part of VFD2/TAS1R2 by ~6 Å and that these changes get transmitted from VFD2 of TAS1R2 (where agonists bind) through the VFD3 and the CRD3 to the TMD3 of TAS1R3 (which couples to the G protein) [113].

Bruno et al. [114] studied the heterodimeric family C/family A mGluR2/5HT_2A_ complex using all-atom molecular dynamics simulations and identified a cross-talk between the two protomers and observed the effect of the heterodimerization on the shape of the binding pocket of 5HT_2A_ receptor.

#### 3.3.2. Family A

The molecular aspects of signal transduction through family A GPCRs dimers have been not well investigated and require further studies. In this context Fanelli and Felline [115] determined that dimerization and ligand binding affect the structure network of adenosine A_2A_ receptor. Jonas et al. [116] reported super-resolution imaging of functionally asymmetric oligomers which reveal diverse functional and structural organizations and the ability to alter signal responses. Navarro et al. [117] used computer modeling, aided by BRET assays to demonstrate molecular architecture formed by a rhombus-shaped adenosine A_1_-A_2A_ receptor heterotetramer, which is bound to two different interacting heterotrimeric G proteins (G_i_ and G_s_). Pediani et al. [118] reported dynamic regulation of quaternary organization of the M_1_ muscarinic receptor by subtype-selective antagonist drugs. Baltoumas et al. [119] and Kaczor et al. [120] used molecular dynamics to study molecular aspects of GPCRs dimerization and dimer-ligands interactions.

#### 3.3.3. The Role of Membrane Cholesterol

There are only a few publications about a key role of cholesterol in GPCR dimerization. Prasanna et al. [121] reported that cholesterol modulates the dimer interface of the β_2_-adrenergic receptor via cholesterol occupancy sites. The same group [122] also found that the presence of cholesterol at the dimer interface is correlated with increased dimer plasticity and flexibility. In this context Pluhackova et al. [123] provided a molecular basis for the modulation of GPCR activity by its lipid environment. They used molecular dynamics simulations to show that CXCR4 dimerizes promiscuously in phospholipid membranes. However, addition of cholesterol dramatically affects the dimerization pattern: cholesterol binding largely abolishes the preferred dimer motif observed for pure phospholipid bilayers formed mainly by transmembrane helices 1 and 7 (TM1/TM5–7) at the dimer interface [123]. Interestingly, the symmetric TM3,4/TM3,4 interface is enabled first by intercalating cholesterol molecules.

## 4. Signaling within Complexes of GPCRs with Other Protein Classes

Classically GPCRs have been considered to transduce signal into intracellular second messengers by functioning as ligand-regulated guanine nucleotide exchange factors for a family of G proteins [124]. It is nowadays well-demonstrated that GPCRs can signal via G-protein-independent mechanisms, e.g., β-arrestin mediated signaling. It is also widely accepted that GPCRs can mediate cell signaling by functioning as scaffolds for the recruitment of either transmembrane or cytosolic GPCR interacting proteins. These proteins, through association with GPCRs, modulate GPCR function and signal transduction.

### 4.1. GRK/Arrestins

GPCR kinases (GRKs) and arrestins were the first proteins identified as GPCR interacting proteins which are engaged in the regulation of GPCR/G-protein coupling. GRKs are important in regulation desensitization of GPCR/G-protein signaling, governing the endocytosis of GPCRs to endosomes to enable GPCR dephosphorylation and resensitization and in GPCR signal transduction via G-protein independent mechanisms [124]. In particular, in accordance with the phenomenon of functional selectivity and biased agonism, there are ligands which can selectively trigger the signal through β-arrestin pathway. As a novel approach, targeting β-arrestin function in the dopaminergic system might be desirable because through its desensitization of G protein signalling, it can reduce dyskinesias and simultaneously through its signaling ability facilitate locomotion, without potentially affecting other neurotransmitter systems. Urs et al. [125] provided evidence supporting the hypothesis that up-regulating β-arrestin-2 expression ameliorates dyskinesias but enhances the therapeutic effects of levodopa. Thus dopamine receptor agonists biased towards β-arrestin recruitment can be an efficient and safer strategy to treat Parkinson’s disease. Recent studies suggest that selective modulation of individual signalling pathways downstream of the D_2_ receptor may lead to safer antipsychotic drugs [126]. Importantly, blockade of β-arrestin recruitment seems to be a shared property of antipsychotics that exhibit either antagonist or partial agonist activity through Gαi/o-cAMP pathways [126]. This suggests that β-arrestin-biased D_2_ antagonists might exhibit unique antipsychotic profiles [126]. In contrast, a study with analogues of the novel antipsychotic aripiprazole suggested that D_2_ ligands with Gαi/o antagonist and β-arrestin agonist activity may have antipsychotic behavioural activity with reduced extrapyramidal side effects in a mouse model [127]. In contrast, there are also biased agonists completely selective over β-arrestin pathway. As an example, compound PZM21, which is an agonist of µ opioid receptor, activates selectively Gi protein pathway and is almost inactive towards β-arrestin pathway [128]. Compound PZM21, in contrast to morphine, does not cause respiratory depression and reinforcing activity.

### 4.2. Receptor-Activity Modifying Proteins (RAMPs)

Receptor activity-modifying proteins (RAMPs) are a crucial example of proteins that interact with GPCRs to modify their function [129]. RAMPs can function as pharmacological switches and chaperones, and they are able to regulate signalling and/or trafficking in a receptor-dependent manner [129]. They were first identified as chaperones which increased the cell surface expression of the calcitonin-like receptor. RAMPs seem to allosterically affect the structure of calcitonin family receptors enabling their terminal glycosylation in the endoplasmic reticulum, and thus facilitating their expression at the cell surface [124]. Moreover, RAMPs modulate the pharmacology of calcitonin family peptides. Three potential mechanisms are possible here. Firstly, RAMPs are able to affect allosterically the structure of calcitonin family receptors which leads to changes in receptor/ligand specificity. Secondly, RAMPs might contribute to the ligand binding site resulting in a fact that various receptor and RAMP combinations can govern ligand binding specificity. Thirdly, RAMP-regulated terminal glycosylation can affect the specificity of calcitonin peptide binding. It has been also reported that RAMPs regulate not only family B but also family C GPCRs as shown for calcium sensing receptor [130].

### 4.3. Regulators of G-Protein Signaling (RGS)

RGS proteins modulates GPCR signal transduction by functioning as GTPase activating proteins (GAP) which accelerate the hydrolysis of GTP bound to the Gα subunit of G_q_ and G_i/o_ leading to their inactivation [124]. Thus, RGS are capable of terminating G-protein-mediated signal transduction after agonist binding. The studies of RGS protein activity indicates that their functions go beyond GAP activity. Importantly, N-terminus of RGS proteins is a possible site of interaction with GPCRs and other signal transduction proteins [124]. Targeting these interactions may lead to new more specific drugs as RGS proteins regulatory mechanisms may be specific for different cellular environments [124].

### 4.4. Homer Proteins

Homer proteins interact with the proline-rich motif of the C-terminus of group I metabotropic glutamate receptors (mGluRs) 1 and 5 [131]. In this way, Homer proteins modulate the expression and localization of mGluR1a and mGluR5. Homer proteins play a key role in the function and development of the nervous system. mGluR1,5-Homer interactions are a crucial link for group I mGluR-induced synaptic plasticity and fragile X mental retardation syndrome [132]. mGluR1-homer interactions are also implicated in schizophrenia, anxiety, and attention deficits [132].

### 4.5. PDZ Proteins

From the multiple GPCR-interacting proteins, postsynaptic density protein of 95 kD, disc large, zona occludens-1 (PDZ) domain-containing proteins seem to be abundant and have similarly been implicated in disease mechanisms [133]. PDZ proteins are crucial for regulating receptor and channel protein localization within synapses and tight junctions and function to scaffold intracellular signalling protein complexes [133].

### 4.6. Calmodulin

Calmodulin has been demonstrated to interact with the third intracellular loop of the µ opioid receptor and thus to reduce both constitutive and agonist-stimulated G-protein coupling [124]. Furthermore, single nucleotide polymorphisms within the G-protein coupling domain of this receptor are connected with altered calmodulin binding and increase basal µ opioid receptor activity [124]. Calmodulin plays also a role in the modulation of PKC-dependent (heterologous) desensitization of 5-HT_1A_ receptor. mGluR5 also possesses a calmodulin binding site in the C-terminus which overlaps a PKC phosphorylation site, and PKC-mediated phosphorylation and calmodulin binding seems to be antagonistic with one another [124]. Calmodulin also interacts with the C-termini of family B receptors. In summary, calmodulin interactions with GPCRs appear to affect both G-protein-dependent and -independent GPCR signalling as well as receptor trafficking [124].

In summary, strategies to either selectively block or promote the formation of GPCR scaffolded complexes may lead to novel drugs targeting GPCR signalling that is independent of G-protein activation. By targeting specific GPCR interactions, it may be possible to design clinically effective drugs which are more specific and selective, thus devoid of side effects connected with classical GPCR targeting drugs.

## 5. Summary and Perspectives

The role of GPCRs in signaling in living organisms is enormous. Consequently, they are in the spotlight of various drug discovery efforts. Traditional GPCR ligands are usually plain orthosteric ligands, which has a number of drawbacks. On the other hand, recent progress in understanding of GPCR function reveals a picture of very advanced molecular machines, capable of receiving various signals and being modulated by various factors, and giving various responses depending on the particular stimuli. This is a unique opportunity for medicinal chemistry, for it gives a chance of design of precisely tailored compounds, capable of tuning the signaling pathways of ones choice to the desired degree.

Today we can draw some conclusions about the role of TM3, TM6 and TM7 in activation and signaling bias. Although TM3 belongs to the more defined core of TM7 bundle and does not easily undergo rearrangements, mutual information calculations indicate that it can participate in signal transmission. Numerous residues at TM7 play essential role in signal transmission, with NPxxY motif and 7.35 residue being mentioned in increasing number of reports. Notably, some of these studies suggest that these residues may be connected by an allosteric pathway.

More and more data supports the assumed great role of allosteric sodium in activation and signaling bias. In particular, recent data suggest that presence of Na^+^ at its allosteric site in the neighborhood of Asp 2.50 is likely to bias the signal toward arrestin signaling. It also affects behavior of internal water, which can be considered an important element of signal propagation itself.

It is known that dimerization affects GPCR signaling. Obviously, binding of another membrane protein by a receptor can be considered as a strong allosteric factor. Unfortunately, the detailed structural data are still insufficient.

A number of recent reports, both computational and experimental, deliver more and more pieces of the puzzle of internal GPCR signaling pathways. In this review, we took attempt to connect them and reveal the picture of signal processing in GPCRs. Although there is still much to learn, particularly in the dimerization/oligomerization field, recent years certainly make the puzzle more and more complete.
